# Prevalence of Overweight and Obesity among Health Sciences Students in the Amazonia Region of Peru

**DOI:** 10.3390/healthcare8040538

**Published:** 2020-12-04

**Authors:** Joseba Rabanales-Sotos, Maritza Evangelina Evangelina Villanueva-Benites, Jesús Jacinto-Magallanes-Castilla, Zoila Esperanza Leitón-Espinoza, Ángel López-González, Jesús López-Torres-Hidalgo

**Affiliations:** 1 Department of Nursing, Physiotherapy and Occupational Therapy, Nursing Faculty, University of Castilla-La Mancha, Campus Univesitario s/n, 02071 Albacete, Spain; joseba.rabanales@uclm.es; 2 Group of Preventive Activities in the University Health Sciences Setting (UCLM), 02071 Albacete, Spain; jesusl@sescam.org; 3 Scientific University of Peru (Universidad Científica del Perú/UCP), Maynas 80300, Peru; maritza.villanueva@unapiquitos.edu.pe (M.E.V.-B.); jesus.magallanes@unapiquitos.edu.pe (J.J.-M.-C.); 4 Nursing Faculty, National University of Trujillo (Universidad Nacional de Trujillo), Trujillo 130101, Peru; zeleiton@unitru.edu.pe; 5 Albacete Faculty of Medicine (UCLM), Castilla-La Mancha Health Service (Servicio de Salud de Castilla-La Mancha/SESCAM), 02071 Albacete, Spain

**Keywords:** health sciences students, obesity, overweight, lifestyles, preventive activities

## Abstract

A cross-sectional study was conducted to evaluate 199 health sciences students in the city of Iquitos. Their socio-demographic characteristics, lifestyle, level of physical activity, type of food, substance abuse, and prevalence of overweight and obesity were ascertained using purpose-validated questionnaires, i.e., the Systematic Alcohol Consumption Interview (Interrogatorio Sistematizado de Consumos Alcohólicos/ISCA), a questionnaire on the frequency of dietary intake (CFCA), the International Physical Activity Questionnaire (IPAQ), and anthropometric measurements. The prevalence of overweight (body mass index (BMI) of 25.0–29.9 kg/m^2^) was 26.5% (95% CI = 19.9–33.0%) and that of obesity (BMI ≥ 30.0 kg/m^2^) was 7.9% (95% CI = 3.8–12.1%). A total of 34.4% of students (95% CI = 27.4–41.4%) presented with a BMI > 25 kg/m^2^. The frequency of overweight was significantly higher in persons aged over 20 years (OR = 2.5) and smokers (OR = 3.2), and the frequency of obesity was significantly higher in older students (OR = 4.1) and males (OR = 5.5). In conclusion, a considerable proportion of health sciences students in the Amazonia region presented with a high BMI. The proportion of students with overweight was higher among students aged over 20 years and smokers, while that of obesity was also higher among males. In the university setting, the development of more overweight- and obesity-preventive activities and educational interventions would therefore be desirable.

## 1. Introduction

In the 21st century, the prevention of chronic non-transmissible diseases is one of the most important public health challenges. According to the World Health Organization (WHO), chronic non-transmissible diseases cause 71% of deaths worldwide, especially in adults aged 30 to 69 years. Over 85% of these deaths occur in low- to middle-income countries, with cardiovascular diseases being the cause of the majority of chronic diseases. It is envisaged that in the coming years, cardiovascular diseases will cause three times more deaths and disabilities than infectious diseases [1]. In Latin America, cardiovascular and cerebrovascular diseases are reckoned to be responsible for 35–55% of all deaths [2].

Non-transmissible diseases progress slowly and affect all age groups. The adolescent and young adult population, such as university students, is also exposed. This age sees the emergence of population risk factors, such as overweight, obesity, smoking, excessive alcohol consumption, and sedentariness [3].

According to WHO data, some 1400 million adults worldwide present with obesity and/or overweight, which constitute significant risk factors for morbidity and mortality [4]. The prevalence of overweight in young adults varies depending on the country, e.g., 16 to 37% in Spain [5], 2.9 to 14.3% in China, 11 to 37.5% in India, around 17% in the USA, and 13.4 to 31.6% in Latin America and South Africa [6].

In Peru, data produced by the demographic and family health survey (Encuesta Demográfica y de Salud Familiar/ENDES) show that 35.5% of persons aged 15 years and over present with overweight, and that obesity affects 18.3% of adolescents, with a higher frequency in females [7]. In the group of adolescents and adults, one in three presents with overweight or obesity [8].

Overweight and obesity is a highly complex chronic condition that develops in the face of potentially hundreds of factors including the consequences of a suboptimal diet that is predominated by high caloric consumption, due mainly to excess lipids and carbohydrates, and is accompanied by low levels of physical activity, all of which generates a lifestyle harmful to health [9].

The transition from adolescence to adult life has been described as a critical period in the development of obesity and less healthy lifestyles. A lack of time, self-discipline, or social support, as well as a lack of parental control, have been described by the university student population as the most important barriers to engaging in healthy activities [10]. A number of previous studies have highlighted certain problems, such as smoking and the consumption of alcohol and other drugs among university students, with the following proportions of hazardous drinkers being reported: 37.1% among Spanish university students [11], 11.9–33% among medical students from other countries [12], and 17.9–27.2% among nursing students in countries such as Spain and Costa Rica [13,14]. Previous studies also highlight the fact that university students, especially those involved in health sciences, adopt sedentary behaviors, remaining seated for a mean studying time of over 8 h per day [15]. Overall, only about 50% of university students do some type of physical exercise [16].

Accordingly, the aim of the study was to ascertain the prevalence of overweight and obesity in health sciences students in Peru’s Amazonia region and establish its relationship with socio-demographic variables, level of physical activity, type of diet, and presence of unhealthy habits.

## 2. Materials and Methods

### 2.1. Design

We carried out a cross-sectional observational descriptive study in which health sciences students at the Scientific University of Peru (PSU) participated during the 2017–2018 academic year. The study inclusion criteria were: registration as a student of nursing, obstetrics, stomatology, psychology, or medical technology during the above academic year, and providing consent to participate. The sole exclusion criterion was the refusal to participate in the study after learning its designated goals.

The study was authorized by the Chancellor and Senate (acting in their capacity as a Research Ethics Committee) of the PSU (09/2916), and the Helsinki Declaration principles were observed at all times. To preserve the confidentiality of the participants, the questionnaire data were entered into a database and identified exclusively by a numerical code.

### 2.2. Study Population

All registered students of Health Sciences, a total of 250, were invited by email to participate in the study. All of them were informed in detail about the nature of this study. A total of 199 students (79.6%) attended the appointment to collect data, answer the survey, and measurement of anthropometric parameters. All participants provided written informed consent. The sample size achieved corresponded to an expected frequency of overweight and obesity of 22% [17], assuming a 95% confidence level and a precision of ±2.6%.

### 2.3. Information Sources

To collect the data, we designed a pre-coded, self-administered, anonymous data collection form, which was completed in the lecture rooms by students in groups of 20 to 25. For the purpose of measuring anthropometric variables (weight, height, and body composition (BC)), the students were asked to go to the university medical center, where they were evaluated over a period of thirty minutes. All data were taken under standard conditions by the researchers. After participating in the study, they were informed about their body composition, as well as unhealthy habits.

### 2.4. Variables

The variables considered in the study were as follows:

(a) Socio-demographic characteristics (age, sex, form of coexistence, place of origin according to number of inhabitants, social class based on parents’ occupation or that of the students themselves, and whether they were engaged in some gainful occupational activity using Goldthorpe’s classification) [18].
(b) Anthropometric measures: weight (average of two determinations measured using a certified Seca-770 scale (SECA gmbh & co. kg, Hamburg, Germany) with easy calibration, with the participant barefoot and in light clothes), height (average of two determinations measured using wall-mounted Seca-222 height rod (SECA gmbh & co. kg, Hamburg, Germany), with the participant standing barefoot on standing position and joining their sagittal average line with the height rod average line), body mass index (BMI) calculated as weight (kg)/height^2^ (m^2^), and BC measured using a Tanita MC 780-P MA^®^ (TANITA Corporation, Tokyo, Japan) segmental body composition monitor.
(c) Level of physical activity, as measured using the Physical Activity Questionnaire (IPAQ) [19], with students being classified as involved in sedentary, moderate, or vigorous physical activity.
(d) Quality of diet: frequency of weekly consumption of each group of foods, with healthy diet criteria being defined as the consumption of 3–4 weekly rations of fish and seafood, lean meat and eggs; 2–4 weekly rations of legumes; 2–4 daily rations of dairy products; 2 or more daily rations of green leafy and other vegetables; 3 or more daily rations of fresh fruit; 4–6 daily rations of bread, cereals, pasta, rice or potatoes [20].
(e) Unhealthy habits: smoking habit, substance abuse, and alcohol consumption. The alcohol consumption was measured using the Systematic Alcohol Consumption Interview (ISCA) [21]. The ISCA consists of three questions that address the quantity and frequency of alcohol consumption, differentiating between workdays and weekends/holidays, and enables quantifying weekly intakes that are deemed to be hazardous by the WHO.

### 2.5. Statistical Analysis

Once the participants’ responses had been entered into a database, they were processed and analyzed. All statistical analyses of the data were performed using the IBM SPSS Statistics V.24 software program (SPSS Inc., Chicago, IL, USA). First, the participants’ characteristics were described, calculating the distribution of frequencies, 95% confidence intervals, and measures of the central trends and dispersions. Thereafter, tests involving the comparison of proportions (likelihood-ratio chi-squared test) and means (Student’s *t*-test), or their non-parametric alternative (Mann–Whitney *U* test), were used to test for independence among the main variables using a significance level of 0.05. Lastly, two logistic regression models were fitted to ascertain the association between the different variables and the presence of overweight and obesity, with these being used as dependent variables and adjustments being made for possible confounding factors. The model was interpreted by determining the statistical significance of the coefficients with the aid of the Wald test and the odds ratios of the explanatory variables.

## 3. Results

A total of 199 students were evaluated, with a mean age of 20.7 years (SD = 5.7 years). Table 1 shows the breakdown of their socio-demographic characteristics.

**Figure 1:**
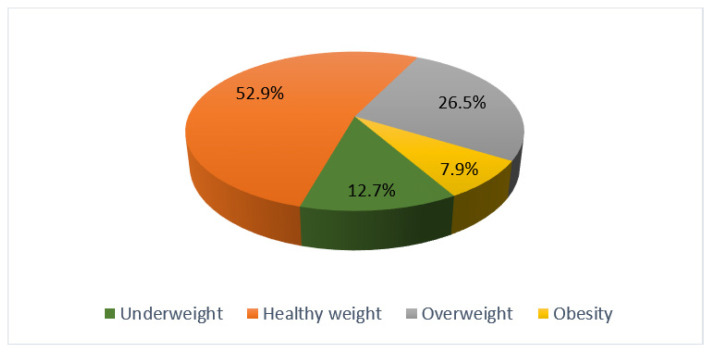
Distribution of students by body mass index (BMI).

Excluding students among whom the BMI could not be ascertained (10 cases), the prevalence of overweight (BMI = 25.0–29.9 kg/m^2^) was 26.5% (95% CI = 19.9–33.0%), and that of obesity (BMI ≥ 30.0 kg/m^2^) was 7.9% (95% CI = 3.8–12.1%) (Figure 1). Overall, 34.4% of students (95% CI = 27.4–41.4%) presented with a BMI > 25 kg/m^2^.

Among the students, the proportion of inactive subjects was 43.7%, the proportion of those doing moderate physical activity was 21.6%, and the proportion of those doing intense activity was 34.7%. The proportion of subjects with overweight or obesity was not significantly different between the active and inactive subjects, though, among those doing intense physical exercise, a higher mean lean body mass value was found (45.6 ± 7.2 kg for students doing intense exercise vs. 43.5 ± 7.1 kg for the rest; *p* = 0.01), as was a higher mean muscle mass value (43.3 ± 6.9 kg for students doing intense exercise vs. 41.3 ± 6.7 kg for the rest; *p* = 0.01).

With respect to unhealthy habits, the proportion of smokers was 12.6%, that of hazardous drinkers was 1.5%, and that of substance abusers was 1.5%. The proportion of smokers was significantly higher among obese subjects (50% vs. 9.8%; *p* < 0.001), and overall among subjects presenting with a high BMI (20.3% vs. 8.9%; *p* = 0.02).

In terms of socio-demographic characteristics (Table 2), while the proportion of students with an under- or overweight status was not significantly different between men and women, the proportion of obese subjects was significantly higher among men (20.0% vs. 5.7%; *p* = 0.01). The mean age in years was higher in both obese (23.9 ± 8.9 SD vs. 20.4 ± 5.4 SD; *p* = 0.02) and overweight subjects (22.7 ± 7.4 SD vs. 20.0 ± 4.9 SD; *p* = 0.01). When it came to forms of coexistence, the proportion of overweight or obese students was significantly lower among those who cohabited with their parents in the family home compared to other forms of coexistence (30.1% vs. 48.8%; *p* = 0.02). No relationship was observed between the presence of overweight or obesity and social class based on occupation or size of the town of origin.

Regarding a healthy diet, the mean compliance with the eight criteria considered was 2.5 (SD = 1.3), with the distribution shown in Table 3 below. Although compliance with the healthy dietary criteria was lower among subjects with a BMI > 25 kg/m^2^, the difference was not statistically significant with respect to those who presented a BMI within normal limits.

The logistic regression (Table 4) showed that the variables associated with the existence of a high BMI (≥25 kg/m^2^) were being aged 20 years or over (OR = 2.5) and a smoking habit (OR = 3.2), while those associated with the presence of obesity (BMI ≥ 30 kg/m^2^) were being aged 20 years or over (OR = 4.0) and the male gender (OR = 5.5).

## 4. Discussion

This study evaluated the BMI of health sciences students in Peru’s Amazonia region, with the aim of describing the proportion of obesity and/or overweight, and the relationship that these may display with other variables, such as the level of physical activity, type of diet, and presence of unhealthy habits. The results show that a high proportion of university students presented with a high BMI. Approximately one-third of students had a BMI > 25 kg/m^2^, with around one in four presenting with overweight and close to 8% presenting with obesity. These results are comparable to those of other studies conducted on Latin American university students [22,23].

A study conducted in Mexico in 2015 by Lorenzini et al. [24] reported a significantly higher proportion of obese students among men. In our study, no statistically significant differences related to sex were observed among students with a BMI ≥ 25 kg/m^2^, which is consistent with other studies that describe a higher prevalence of overweight among women [25,26].

Physical activity is beneficial at both the physical and psychological levels, improves body composition, and reduces the development of metabolic diseases [27,28]. Despite the benefits demonstrated by physical activity, our results show, like others, that a high proportion of youth were inactive or sedentary subjects [28,29,30]. However, in our results and in those of other previous studies [31], the proportion of overweight or obesity was not significantly different between active or inactive students.

It is during the university stage that young adults usually first assume responsibility for their diet, and indeed, they have been described as a vulnerable group from a nutritional standpoint [32,33,34]. Despite the fact that our study subjects were health sciences students, this group has been reported as having a dietary imbalance caused by a high intake of nutrients, with refined carbohydrates, simple sugars, and saturated fats in particular, as well as a low intake of fruit and green leafy vegetables [28,34,35]. In our case, even though compliance with healthy dietary criteria was lower among subjects with a high BMI, the difference did not prove to be statistically significant vis-à-vis those who had a BMI within the normal limits.

The university setting increases the risk of developing a smoking habit, and our study found a heavier smoking habit among individuals who presented with a high BMI. The proportion of student smokers was approximately 13%, which is a figure that is very similar to that reported for nursing students in Colombia [36]. Nonetheless, this proportion is lower than that observed in university students drawn from other disciplines and countries [37,38,39]. On comparing the prevalence in both sexes, no significant difference was observed between men and women, a finding that is in line with the results of other studies conducted on university students [38,40], though there is no unanimity in the references consulted since higher tobacco use has occasionally been reported in males [36]. With respect to alcohol, only 1.5% of students displayed a level of consumption that is considered hazardous. This result reveals a low proportion of consumption, which is a finding that is not reported in other studies conducted on university students from different countries [41,42].

Universities are ideal scenarios for creating health and wellbeing promotion settings and for implementing lifestyle improvement strategies [43]. It is important to implement programs that include interventions that are designed to increase students’ physical activity levels and reduce sedentariness [30]. These programs should also provide educational activities that focus on healthy eating habits and target reducing the consumption of fats, carbohydrates, and sugars. Stress should also be laid on the need to design interventions aimed at the prevention of alcohol consumption and smoking in the university setting. Previous experiences show satisfactory results, though it is suggested that studies should be undertaken on larger populations [44,45].

In general, risk behaviors, such as smoking and alcohol consumption, lack of physical activity, and suboptimal diet, as well as their consequences, including overweight and obesity, are important public health problems. In the future, new studies should be undertaken that ascertain which risk factors may be modifiable when it comes to preventing cardiovascular diseases and other health problems.

To date, it has been established that certain socio-demographic characteristics and habits are associated with alterations in BMI, yet knowledge in this area is limited since most research has been carried out on specific groups and there may be important differences in different cultural settings [46,47,48]. Furthermore, the references consulted on obesity and/or overweight among university students identify gaps in the knowledge [49,50,51,52] since there has been no uniform methodology in the evaluations performed and it is not a problem that is studied in depth in all countries or specific regions, as is the case in the Amazonia region of Peru.

Our data should be considered with caution since there was a large difference in the size of the participant groups (33 men and 166 women) and they were not randomly selected subjects; thus, the conclusions might be affected by the volunteer bias.

Although a high response rate was obtained, when it comes to studying limitations, it should be noted that our results might underestimate overweight/obesity if classroom attendance proves to be related to healthier lifestyles. There could also be biases in the results if non-attendance were proven to be more frequent among persons with different socio-demographic characteristics and work obligations. With respect to the students’ degree of truthfulness in their replies, this can be viewed as high in view of the fact that questionnaire completion was both voluntary and anonymous. It is important to recognize that there is also the risk of a socially desirable response bias.

## 5. Conclusions

In conclusion, one-third of all health sciences students in Amazonia presented with a BMI of 25 kg/m^2^ or higher. While the proportion of overweight students was higher in those aged over 20 years and in smokers, the proportion of obesity was higher in subjects aged over 20 years and in males. In the university setting, the development of more overweight- and obesity-preventive activities and educational interventions would therefore be desirable, especially if the target subjects are future health professionals, who should ideally rank as a standard for healthy habits in any given society.

## Figures and Tables

**Table 1:** Socio-demographic characteristics of the students.

Characteristics	No.	%
Sex		
Men	33	16.6
Women	166	83.4
Age		
18 to 19 years	118	59.3
20 to 24 years	50	25.1
25 years or over	31	15.6
Qualification		
Nursing	37	18.6
Obstetrics	50	25.1
Stomatology	45	22.6
Psychology	47	23.6
Medical technology	18	9.0
No data	2	1.0
Academic year		
First	94	47.2
Second	96	48.2
Third	9	4.5
Origin		
Town of fewer than 10,000 inhabitants	28	14.0
Town of 10,000 to 40,000 inhabitants	20	10.1
Town of over 40,000 inhabitants	146	73.4
No data	5	2.5
Form of coexistence		
Lives alone	20	10.1
Lives with a partner (with or without children)	19	9.5
Lives with parents (with or without siblings)	154	77.4
Other forms of coexistence	6	3.0
Social class *		
I	25	12.6
II	55	27.6
III (a,b)	31	15.6
IV (a,b,c)	25	12.6
V–VII (a,b)	39	19.5
No data	24	12.1

* Social class: Type I: Higher-grade professionals, administrators, and officials; managers in large industrial establishments; large proprietors. Type II: Lower-grade professionals, administrators, and officials; higher-grade technicians; managers in small industrial establishments; supervisors of non-manual employees. Type IIIa: Routine non-manual employees, higher grade (administration and commerce). Type IIIb: Routine non-manual employees, lower grade (sales and services). Type IVa: Small proprietors, artisans, etc., with employees. Type IVb: Small proprietors, artisans, etc., without employees. Type IVc: Farmers, smallholders, and other self-employed workers in primary production. Type V: Lower-grade technicians and supervisors of manual workers. Type VI: Skilled manual workers. Type VIIa: Semi-skilled and unskilled manual workers (not in agriculture or other forms of primary production). Type VIIb: Agricultural and other workers in primary production.

**Table 2:** Level of physical activity, healthy dietary criteria, and socio-demographic characteristics of the students that presented a BMI within normal limits vs. a high BMI.

Variables	BMI < 25 kg/m^2^ No. (%)	BMI ≥ 25 kg/m^2^ No. (%)	*p*
Level of physical activity			0.551
Inactive	58 (46.8)	26 (40.0)	
Moderate	26 (21.0)	13 (20.0)	
Intense	40 (32.3)	26 (40.0)	
Healthy dietary criteria			0.889
2 criteria or fewer	52 (52.5)	29 (53.7)	
3 criteria or over	47 (47.5)	25 (46.3)	
Sex			0.481
Men	18 (14.5)	12 (18.5)	
Women	106 (85.5)	53 (81.5)	
Form of coexistence			0.023
Live with partner	102 (82.3)	44 (67.7)	
No live with partner	22 (17.7)	21 (32.3)	
Origin			0.845
Town of fewer than 40,000 inhabitants	28 (23.3)	16 (24.6)	
Town of over than 40,000 inhabitants	92 (76.7)	49 (75.4)	

**Table 3:** Description of compliance with healthy dietary criteria among the students.

Healthy Dietary Criteria	Men No. (%)	Women No. (%)	Total No. (%)	*p*
Consumption of 3–4 weekly rations of fish and seafood				
Yes	7 (21.2)	32 (19.3)	39 (19.6)	0.797
No	25 (75.8)	129 (77.7)	154 (77.4)	
No data	1 (3.0)	5 (3.0)	6 (3.0)	
Consumption of 3–4 weekly rations of lean meat				
Yes	9 (27.3)	36 (21.7)	45 (22.6)	0.518
No	24 (72.7)	127 (76.5)	151 (75.9)	
No data	0 (0.0)	3 (1.8)	3 (1.5.5)	
Consumption of 3–4 weekly rations of eggs				
Yes	9 (27.3)	50 (30.1)	59 (29.6)	0.682
No	24 (72.7)	112 (67.5)	136 (68.3)	
No data	0 (0.0)	4 (2.4)	4 (2.0)	
Consumption of 2–4 weekly rations of legumes				
Yes	11 (33.3)	58 (34.9)	69 (34.7)	0.840
No	21 (63.6)	102 (61.4)	123 (61.8)	
No data	1 (3.0)	6 (3.6)	7 (3.5)	
Consumption of 2–4 daily rations of dairy products (milk, cheese, yogurt)				
Yes	11 (33.3)	61 (36.7)	72 (36.2)	0.744
No	21 (63.6)	102 (61.4)	123 (61.8)	
No data	1 (3.0)	3 (1.8)	4 (2.0)	
Consumption of ≥2 daily rations of green leafy and other vegetables				
Yes	9 (27.3)	33 (19.9)	42 (21.1)	0.370
No	24 (72.7)	130 (78.3)	154 (77.4)	
No data	0 (0.0)	3 (1.8)	3 (1.5)	
Consumption of ≥3 daily rations of fresh fruit				
Yes	3 (9.1)	24 (14.5)	27 (13.6)	0.490
No	30 (90.9)	130 (78.3)	160 (80.4)	
No data	0 (0.0)	12 (7.2)	12 (6.0)	
Consumption of 4–6 daily rations of bread, cereals, pasta, rice, and potatoes				
Yes	25 (75.8)	112 (67.5)	137 (68.8)	0.421
No	8 (24.2)	51 (30.7)	59 (29.6)	
No data	0 (0.0)	3 (1.8)	3 (1.5)	

**Table 4:** Variables that were found by logistic regression to be associated with the presence of a high BMI.

Variables	Variables	B	Wald	*p*	OR (95% CI)
BMI > 25 kg/m^2^	Age ≥ 20 years vs. age ≤ 20 years	0.923	7.788	0.005	2.5 (1.3–4.8)
	Smoking habit vs. no smoking habit	1.148	4.843	0.028	3.2 (1.3–8.8)
	Active participant vs inactive participant	0.320	0.949	0.330	1.4 (0.7–2.6)
	Healthy diet vs. unhealthy diet	0.108	0.223	0.637	1.1 (0.7–1.7)
	Man vs. woman	0.042	0.08	0.928	1.0 (0.4–2.6)
Obesity (BMI ≥ 30 kg/m^2^)	Age ≥ 20 years vs. age ≤ 20 years	1.391	5.287	0.021	4.1 (1.2–13.1)
	Smoking habit vs. no smoking habit	0.225	0.388	0.534	1.253 (0.616–2.546)
	Active participant vs inactive participant	0.088	0.023	0.878	1.1 (0.3–3.3)
	Healthy diet vs. unhealthy diet	0.140	0.131	0.718	0.8 (0.4–18)
	Man vs. woman	1.7120	7.836	0.005	5.5 (1.7–18.3)

The independent variables that were included in the models were age, smoking habit, active participant, healthy diet, and sex, adjusted by BMI.
